# Combined perventricular closure of ventricular septal defect and atrial septal defect via lower ministernotomy

**DOI:** 10.1186/s13019-018-0815-5

**Published:** 2018-12-12

**Authors:** Yunfei Ling, Yue Wang, Qiang Fan, Yongjun Qian

**Affiliations:** Department of Cardiovascular Surgery, West China Hospital, Sichuan University, Guoxuexiang 37th, 610041 Chengdu, Sichuan People’s Republic of China

**Keywords:** Ventricular septal defect, Atrial septal defect, Perventricular closure

## Abstract

**Background:**

Minimally invasive approaches such as perventricular closure of ventricular septal defects (VSD) have been applied for the surgical correction of congenital heart defects in order to avoid disadvantages related to median sternotomy with a cardiopulmonary bypass (CPB). However, reports remain scarce regarding combined perventricular closure of VSD and atrial septal defects (ASD) via minimally invasive approaches, such as lower ministernotomy.

**Results:**

The authors have operated on 5 patients who were diagnosed with VSD in association with ASD, successfully realizing perventricular closure via lower ministernotomy.

**Conclusion:**

The proposed technique proved to be safe and effective.

## Background

Ventricular septal defects (VSD) are some of the most frequent congenital heart anomalies, estimated to account for 20–30% of all congenital cardiac malformations [1, 2]. In the last decade, perventricular approaches have been successfully applied for the closure of various types of VSD, with promising preliminary results [3–5]. However, to date, no reports are available regarding the combined perventricular closure of VSD and atrial septal defects (ASD) via minimally invasive approaches, such as lower ministernotomy.

## Methods

We studied five patients (two boys and three girls) diagnosed with VSD and ASD who underwent operation at the West China Hospital. Answer: We have added the content as follow in the manuscript: All the patients were perimembranous VSD less than 8 mm that was not adjacent to the conduction bundle and aortic valves and the secundum ASD less than 15 mm. Table 1 presents the baseline data of patients.

**Table 1:** Baseline data of patients

Patient NO	Age (month)	Weight (kg)	VSD (mm)	ASD (mm)	VSD occluder (mm)	ASD occluder (mm)
1	19	12	4	6	6	12
2	14	10	5	5	7	12
3	26	13	6	8	8	14
4	18	12.	5	7	7	14
5	20	12	4	10	7	16

*VSD* ventricular septal defect, *ASD* atrial septal defect

**Fig. 1:**
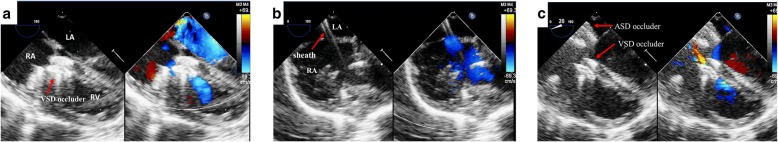
**a** Perventricular VSD closure under TEE guidance. **b** The 12F sheath was positioned across the ASD with its tip in the left atrium. **c** ASD occulder was employed under TEE guidance

After adequate pre-oxygenation, the procedure was performed under general anaesthesia with a standby cardiopulmonary bypass (CPB) machine available. Following a skin incision of approximately 4–5 cm, a partial lower ministernotomy was performed. After surgical exposure of the anterior free wall of the right ventricle, the optimal puncture site was selected per the routine method. Next, we performed perventricular VSD closure under transoesophageal echocardiographic (TEE) guidance using SQFDQ II and SQFDQ IV occlusion devices (MemoPart VSD Occlusion Device; Lepu Medical Technology, Shanghai Shape Memory Alloy Co., Ltd., Shanghai, China) (Fig. 1a), as described in previous reports [6–8]. Then, a 20 G needle was introduced into the right ventricle, and directed up towards the ASD. Then, a 0.025-in. straight-tipped guide wire was introduced through the needle and maneuvered across the tricuspid valve and ASD into the left atrium. Next, the needle was exchanged with a short 12 F sheath over the wire (Fig. 1b). Finally, the sheath was positioned across the ASD with its tip in the left atrium, and the ASD occluder was placed (MemoPart ASD Occlusion Device; Lepu Medical Technology, Shanghai Shape Memory Alloy Co., Ltd., Shanghai, China) (Fig. 1c).

## Results

Surgery was successful in all patients, with no in-hospital mortality or complications. Mean ‘skin-to-skin’ time was 62 ± 31.6 min. No blood infusions or inotropic support were needed in any patient. Mean ventilation support time was 4.2 ± 6.7 h. No rhythm or atrioventricular conduction disorders occurred. No sternal or rib deformations were observed at the time of discharge, and the small skin incision yielded excellent cosmetic results. Mean hospitalization time was 6.5 ± 2.8 days.

## Discussion

Conventionally, most congenital heart defects are treated using CPB via median sternotomy. With the development of percutaneous interventional techniques, various simple congenital structural heart defects may be repaired with comparable results, possibly providing advantages over conventional surgery in some cases [9]. However, transcatheter shunt closure does not appear to be optimal when the patients present other congenital heart diseases. This new technique should be applied with caution in some subtypes of VSDs like perimembranous VSDs adjacent to the conduction bundle and aortic valves and subarterial VSDs, We did not recommend the eccentric occluder device in the perimembranous VSDs; When the ASDs are too small, the angle through the fourth intercostal space to the ASDs will be hard, another left fourth intercostal space will be recommended to close the ASDs. With improvements in device closure techniques, perventricular device closure of VSD under guidance of transesophageal echocardiography (TEE) has been successfully applied in the surgical correction of congenital defects, with promising preliminary results [4]. Based on this technique, we proposed a new approach for combined perventricular closure of VSD and ASD via lower ministernotomy.

## Conclusions

Compared with conventional surgical repair or femoral transcatheter shunt closure, the advantages of this procedure are obvious. It appears to be safe, associated with less operative trauma, and does not require CPB, fluoroscopy or contrast. In selected patients, perventricular device closure of VSD complicated with ASD is effective, and may either substitute or complement conventional surgical closure.

## Abbreviations

ASD: Atrial septal defect
CPB: Cardiopulmonary bypass
TEE: Transesophageal echocardiography
VSD: Ventricular septal defect

## References

[1] Mitchell SC, Korones SB, Berendes HW (1971). Congenital heart disease in 56,109 births. Incidence and natural history. Circulation.

[2] Yang J, Yang L, Yu S (2014). Transcatheter versus surgical closure of Perimembranous ventricular septal defects in children : a randomized controlled trial. J Am Coll Cardiol.

[3] Bacha EA, Cao QL, Starr JP (2003). Perventricular device closure of muscular ventricular septal defects on the beating heart: technique and results. J Thorac Cardiovasc Surg.

[4] Gan C, An Q, Lin K (2008). Perventricular device closure of ventricular septal defects: six months results in 30 young children. Ann Thorac Surg.

[5] Thakkar B, Patel N, Shah S (2012). Perventricular device closure of isolated muscular ventricular septal defect in infants: a single Centre experience. Indian Heart J.

[6] Voitov A, Omelchenko A, Gorbatykh Y, Zaitsev G, Arkhipov A, Soynov I (2017). Outcomes of perventricular off-pump versus conventional closure of ventricular septal defects: a prospective randomized study. Eur J Cardiothorac Surg.

[7] Omelchenko A, Gorbatykh Y, Voitov A, Zaitsev G, Bogachev-Prokophiev A, Karaskov A (2016). Perventricular device closure of ventricular septal defects: results in patients less than 1 year of age. Interact Cardiovasc Thorac Surg.

[8] Yin S, Zhu D, Lin K, An Q (2014). Perventricular device closure of congenital ventricular septal defects. J Card Surg.

[9] Mavroudis C, Backer CL (2013). Pediatric cardiac surgery.

